# Phenotypic Transition From Danon Disease to Arrhythmogenic Cardiomyopathy: Multimodality and Histopathologic Evidence

**DOI:** 10.1016/j.jaccas.2026.110385

**Published:** 2026-09-23

**Authors:** Natália Quintella Sangiorgi Olivetti, Giovana Pereira Belitardo, Felipe Cerqueira Matheus, Deborah Belfort, Luiz Alberto Benvenuti, Luciana Sacilotto, Edimar Bocchi, Giovanna Napolitano, José Eduardo Krieger, Silvia Ayub

**Affiliations:** a Instituto do Coração (InCor), Hospital das Clínicas HCFMUSP, Faculdade de Medicina, Universidade de São Paulo, Sao Paulo, Brazil; b Hospital Israelita Albert Einstein, Sao Paulo, Brazil

**Keywords:** fibrosis, genetic disorders, imaging, systolic heart failure, ventricular tachycardia

## Abstract

**Background:**

Pathogenic variants in lysosome-associated membrane protein 2 (*LAMP2*) gene cause X-linked Danon disease, associated with hypertrophic cardiomyopathy. Arrhythmogenic cardiomyopathy is characterized by myocardial fibrofatty replacement and ventricular arrhythmias.

**Case Summary:**

A 30-year-old woman presented with a hypertrophic phenotype and a short PR interval. At 40 years of age, she developed progressive diffuse low QRS complex voltages and recurrent ventricular arrhythmias. Cardiac magnetic resonance showed extensive ring-like late gadolinium enhancement and biventricular systolic dysfunction, consistent with biventricular arrhythmogenic cardiomyopathy. Genetic testing identified a pathogenic *LAMP2* variant (*LAMP2*:c.928G>A; p.Val310Ile) associated with Danon disease. Despite optimized medical therapy, she progressed to advanced heart failure requiring heart transplantation. The explanted heart demonstrated severe fibrofatty myocardial replacement, with thinning of the right ventricular wall and vacuolated cardiomyocyte, which were strongly stained by periodic acid–Schiff.

**Discussion:**

Danon disease is characterized by myocardial hypertrophy, but not by fibrofatty replacement, which is a hallmark of arrhythmogenic cardiomyopathy. This case demonstrates a rare phenotypic evolution in *LAMP2* cardiomyopathy.

## Visual Summary

**Figure:**
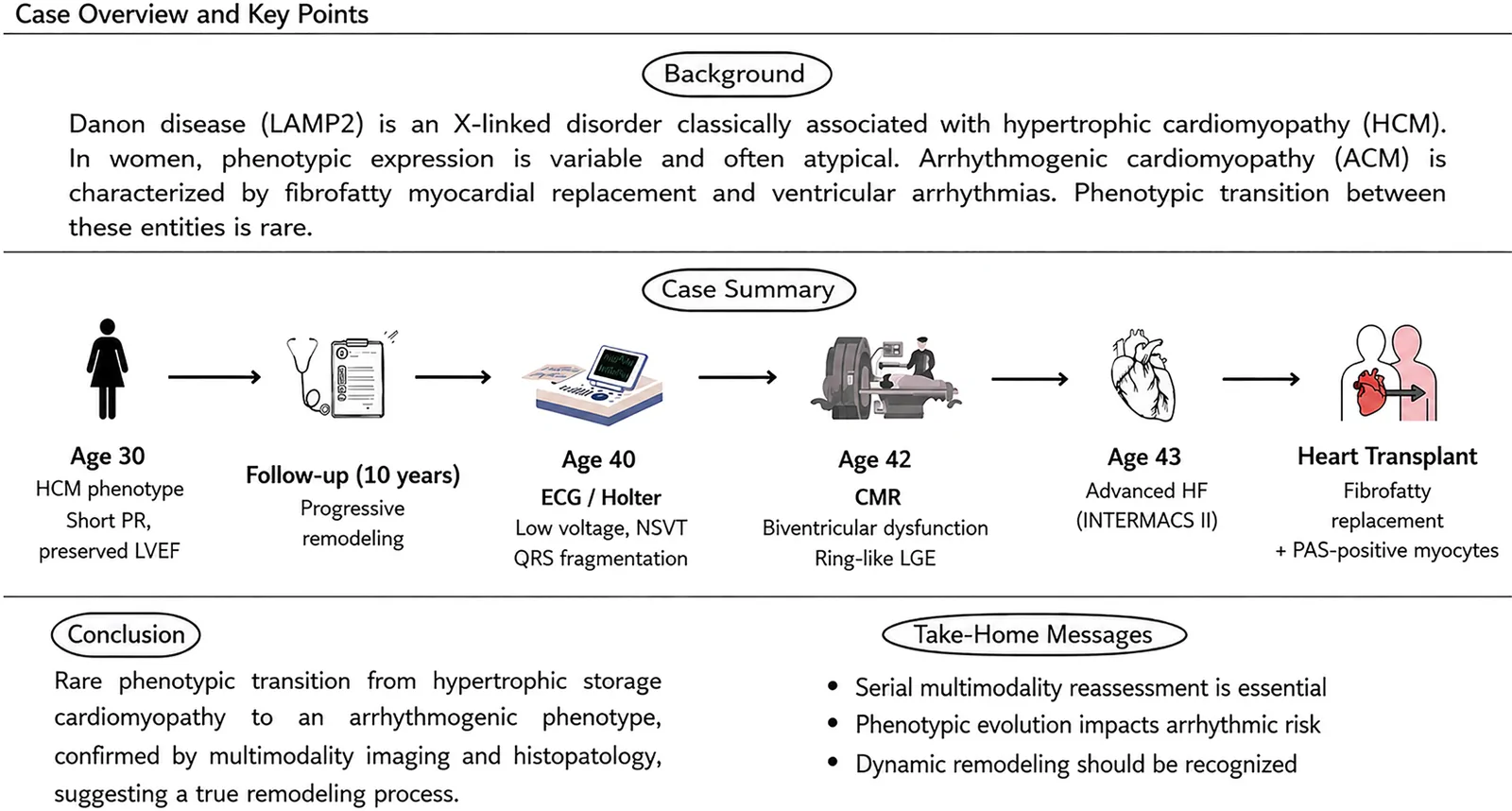

## History of Presentation

A 43-year-old woman was referred to a Precision Medicine Cardiology Program for genetic evaluation of her cardiomyopathy.

## Past Medical History

The patient first underwent cardiac evaluation at 30 years of age. Electrocardiography (ECG) demonstrated sinus rhythm, a short PR interval, and repolarization abnormalities (Figure 1A). Echocardiogram showed mild ventricular hypertrophy (Figure 1B). Cardiac magnetic resonance (CMR) at 31 years of age showed mild septal hypertrophy (maximum wall thickness 13 mm), preserved left ventricular ejection fraction (LVEF) of 61%, and nonischemic late gadolinium enhancement (LGE). The initial phenotype was suggestive of hypertrophic cardiomyopathy. This raised suspicion for an underlying storage disorder based on the combination of concentric left ventricular hypertrophy, short PR interval, and CMR showing diffuse ventricular wall thickening and midmyocardial LGE involving all ventricular segments. No extracardiac manifestations such as skeletal myopathy, visual impairment, or cognitive deficits were noted. Family history was negative for cardiomyopathy or sudden cardiac death. At 40 years of age, she developed progressive diffuse low QRS complex voltages and recurrent ventricular arrhythmias. Electrocardiograms evolved to diffuse low QRS complex voltages, QRS complex fragmentation, and delayed depolarization in lead V_1_ (Figure 2A). Holter monitoring revealed a high ventricular arrhythmic burden (12%) with 205 episodes of nonsustained ventricular tachycardia. She subsequently presented with a sustained ventricular arrhythmia (Figure 2B). The LVEF was 25% at that time. At age 42 years, CMR, performed after the sustained ventricular arrhythmia (Figure 3), demonstrated severe biventricular dysfunction, extensive midmyocardial and subepicardial ring-like LGE and segmental abnormalities involving the inferior left ventricular wall (akinesia), with LVEF 21% and a right ventricular ejection fraction of 40% (Figure 3A). Chest radiography demonstrated remarkable cardiomegaly (Figure 3B). The phenotype fulfilled the Padua criteria for biventricular arrhythmogenic cardiomyopathy (ACM) by exhibiting morpho-functional ventricular abnormalities, global biventricular systolic dysfunction, structural abnormalities characterized by extensive ring-like LGE, repolarization abnormalities with T-wave inversion from V_1_ to V_6_, depolarization abnormalities with diffuse low QRS complex voltages in all limb leads, and ventricular arrhythmias manifested as sustained ventricular tachycardia with a right bundle branch block morphology.

**Figure 1:**
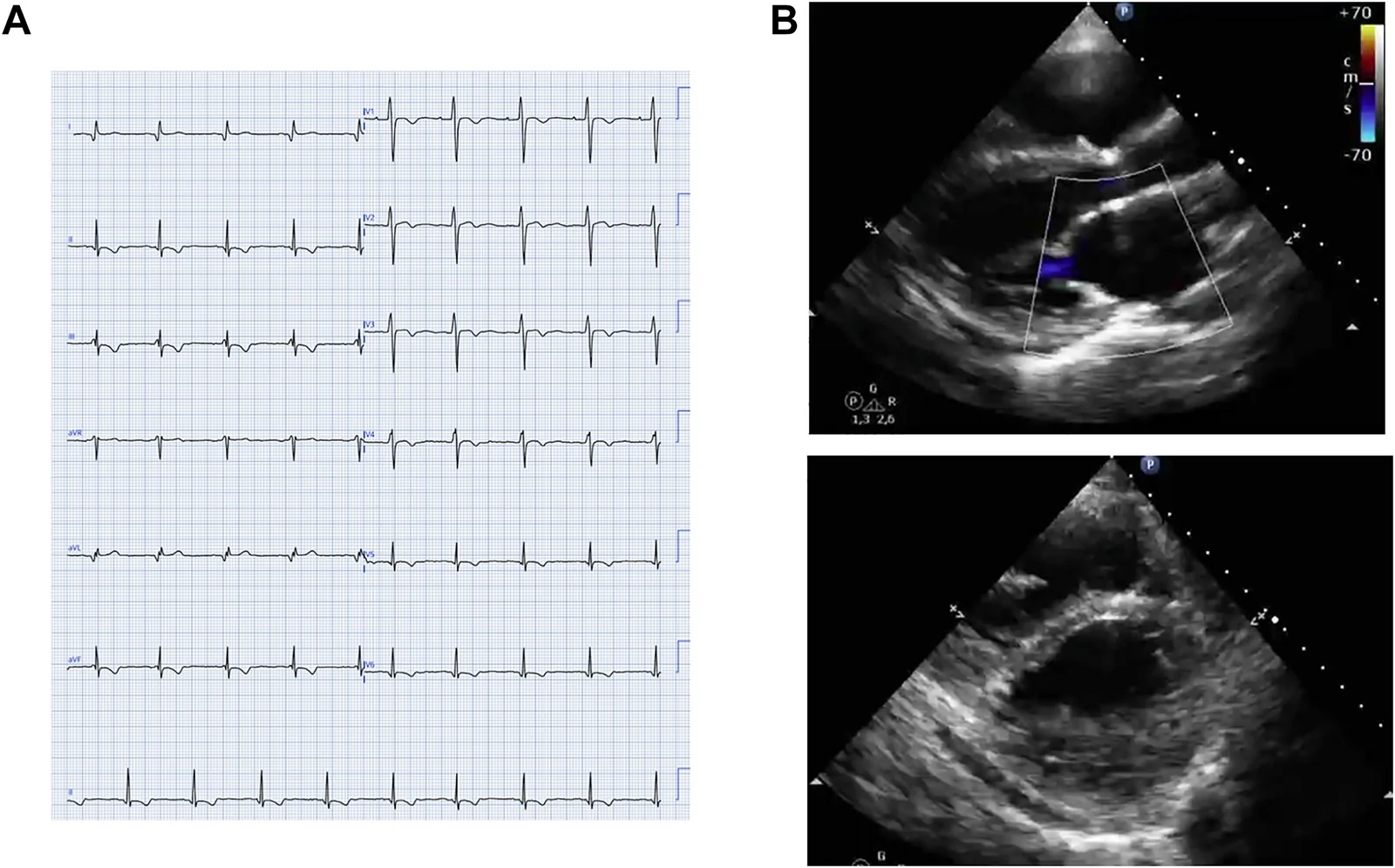
Danon Disease Phenotype: Electrocardiogram and Echocardiogram Features (A) Electrocardiogram demonstrated sinus rhythm, a short PR interval, and repolarization abnormalities. (B) Echocardiogram showed mild ventricular hypertrophy.

**Figure 2:**
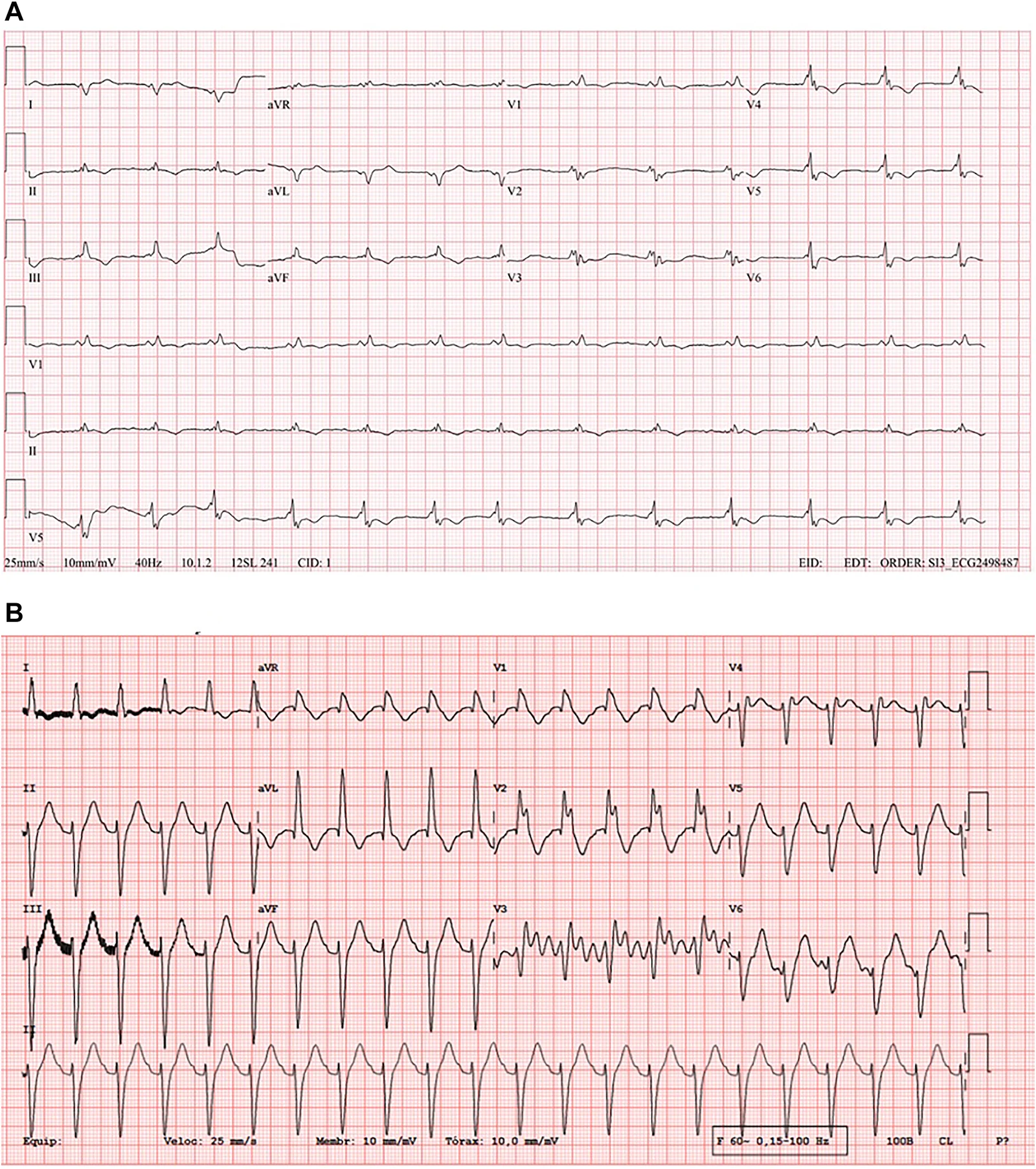
Arrythmogenic Cardiomyopathy Phenotype: Electrocardiogram and Echocardiogram Features (A) Electrocardiograms evolved to diffuse low QRS complex voltages, QRS complex fragmentation, and delayed depolarization in lead V_1_. (B) Sustained ventricular arrhythmia with right bundle branch block morphology and superior axis.

**Figure 3:**
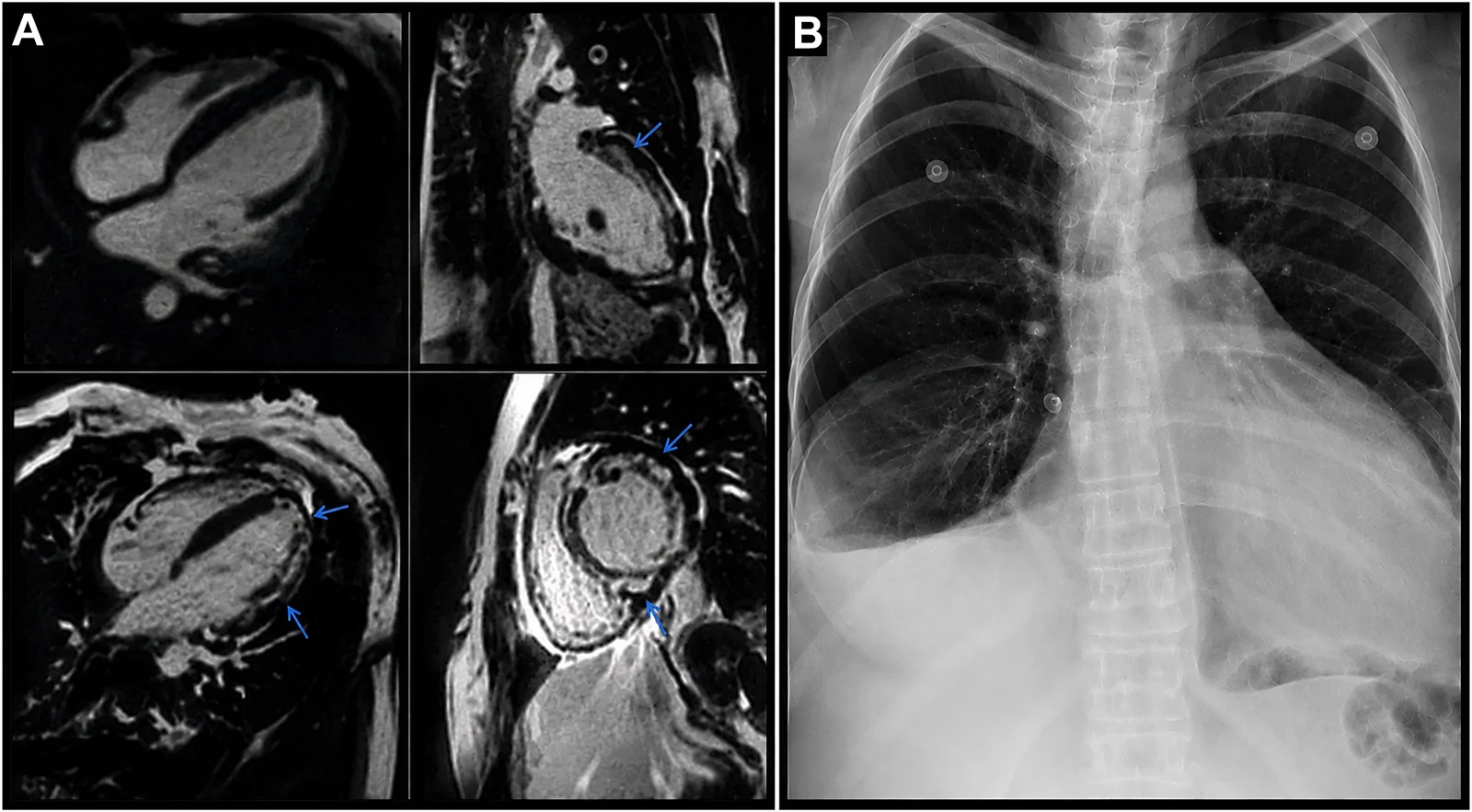
Danon Disease Phenotype: Electrocardiogram and Echocardiogram Features (A) Cardiac magnetic resonance demonstrated severe biventricular dysfunction, extensive midmyocardial and subepicardial ring-like late gadolinium enhancement. (B) Chest radiography demonstrated remarkable cardiomegaly.

## Differential Diagnosis

At first presentation, differential diagnoses included sarcomeric hypertrophic cardiomyopathy, lysosomal storage disorders (including Danon disease and Fabry disease), glycogen storage cardiomyopathy (PRKAG2 syndrome), and infiltrative or inflammatory cardiomyopathies. The subsequent development of biventricular dysfunction, high arrhythmic burden, and extensive nonischemic LGE raised suspicion for ACM.

## Investigations

Genetic testing identified a pathogenic variant in the lysosome-associated membrane protein 2 *LAMP2* (NM_002294.3:c.928G>A; p.Val310Ile**)**, which the lysosome associated membrane protein 2, confirming Danon disease.

## Management

The patient received optimized guideline-directed medical therapy for heart failure, including sacubitril/valsartan, beta-blocker, mineralocorticoid receptor antagonist, and SGLT2 inhibitor. A cardioverter-defibrillator was implanted during follow-up for primary prevention before heart transplantation (HTX).

## Clinical Outcome and Follow-Up

Despite optimized medical therapy, by 43 years of age, she progressed to advanced heart failure, marked by recurrent hospitalizations for decompensation and inotropic dependence. She required continuous dobutamine infusion and intra-aortic balloon pump support. The patient was classified as Interagency Registry for Mechanically Assisted Circulatory Support profile II and subsequently underwent bicaval orthotopic HTX. Pathological examination of the explanted heart demonstrated multiple foci of fibrofatty myocardial replacement in the subepicardial region of both ventricles (Figure 4), most severe in the right ventricle, with thinning of the right ventricular wall, confirming an ACM phenotype. However, there were also multiple foci of enlarged, vacuolated cardiomyocytes, which were strongly stained by periodic acid–Schiff, supporting the diagnosis of lysosomal storage disease. (Figure 5).

**Figure 4:**
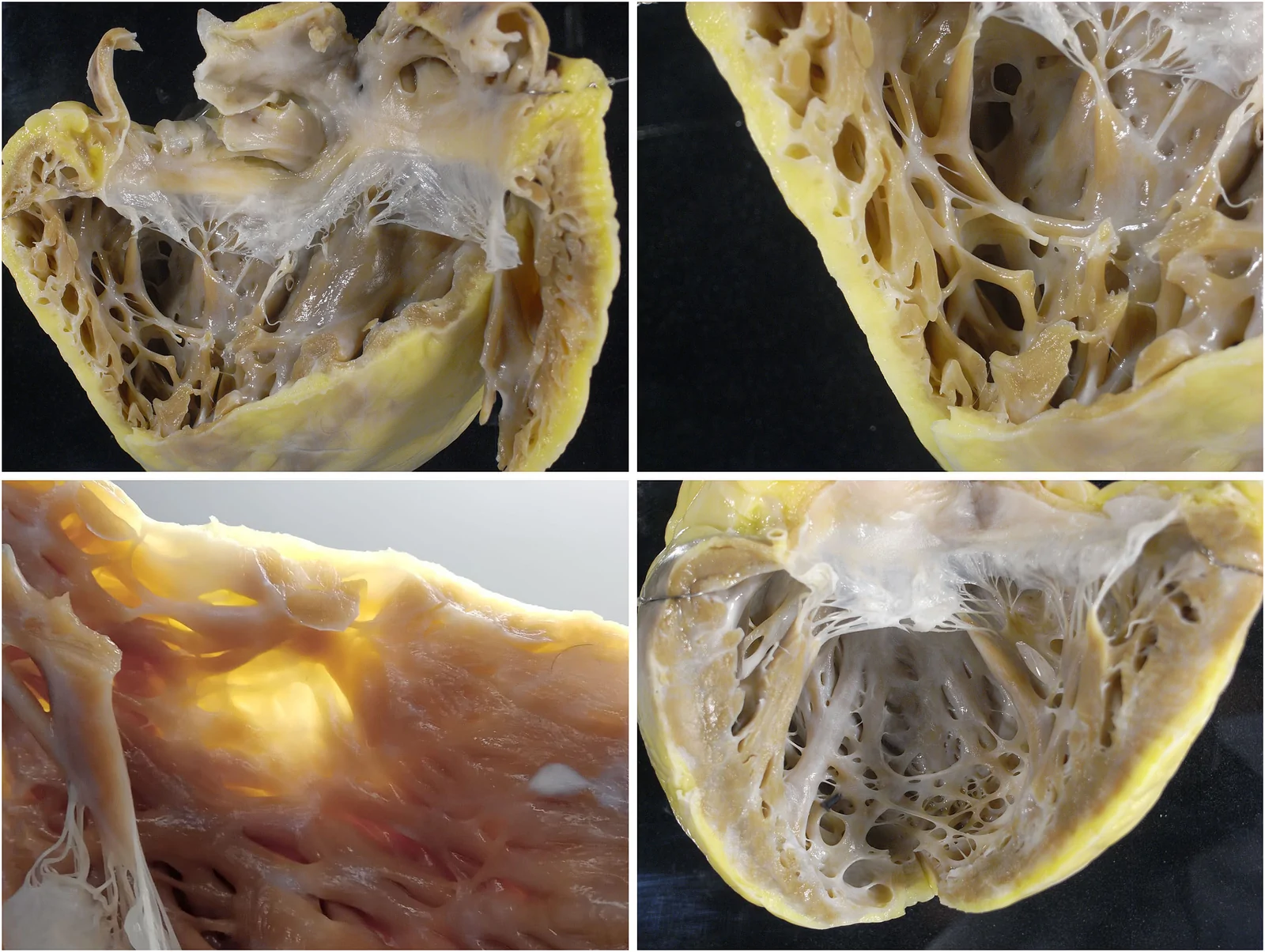
Pathology of the Explanted Heart Demonstrated Thinning of the Right Ventricular Wall, Multiple Foci of Fibrofatty Myocardial Replacement of Subepicardial Region of Both Ventricles, Most Severe in the Right Ventricle

**Figure 5:**
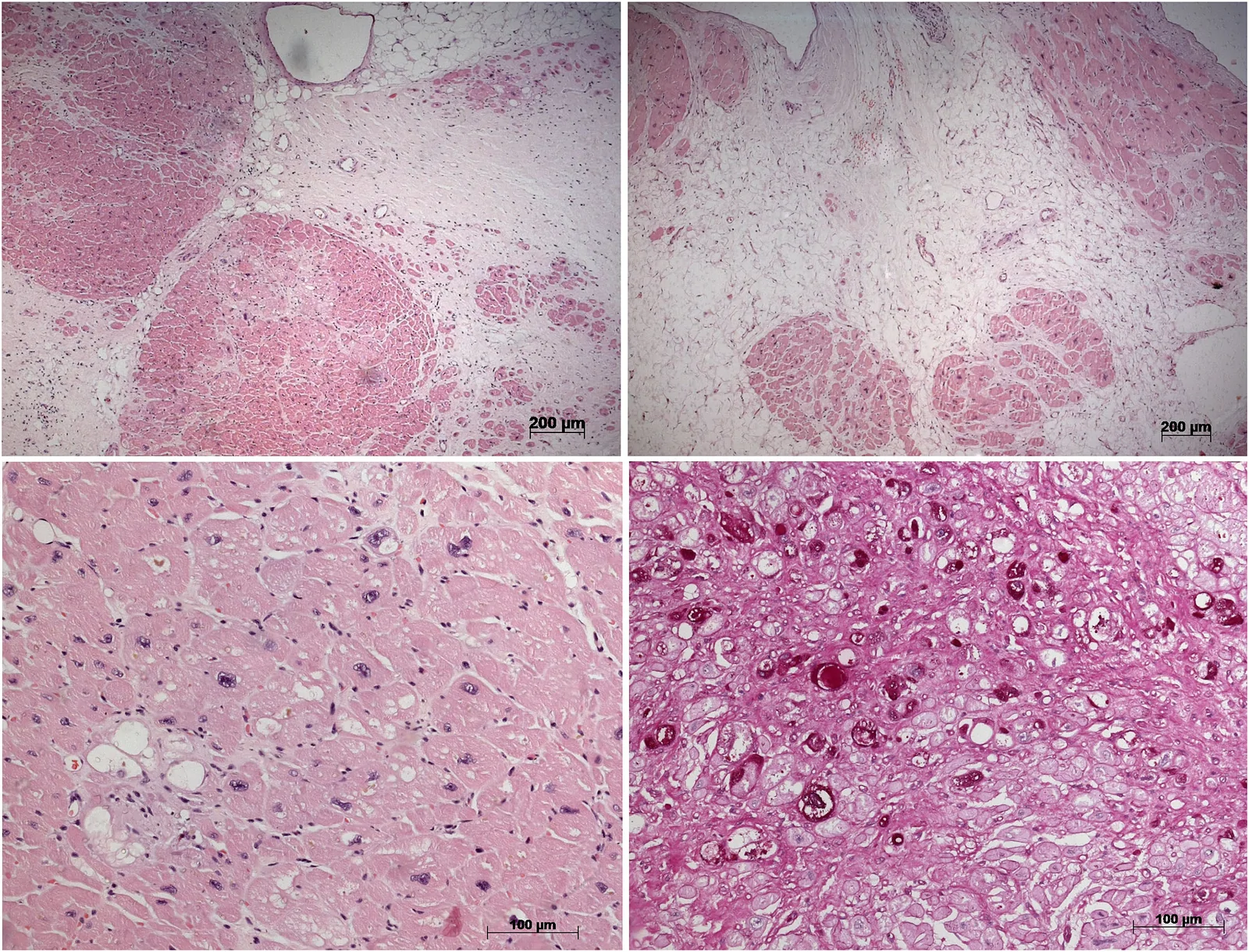
Histopathology of the Explanted Heart Demonstrated Fibrogordurous Substitution (Above) and Multiple Foci of Enlarged, Vacuolated Cardiomyocytes, Which Were Strongly Stained by Periodic Acid–Schiff (Below)

**Visual Summary:**
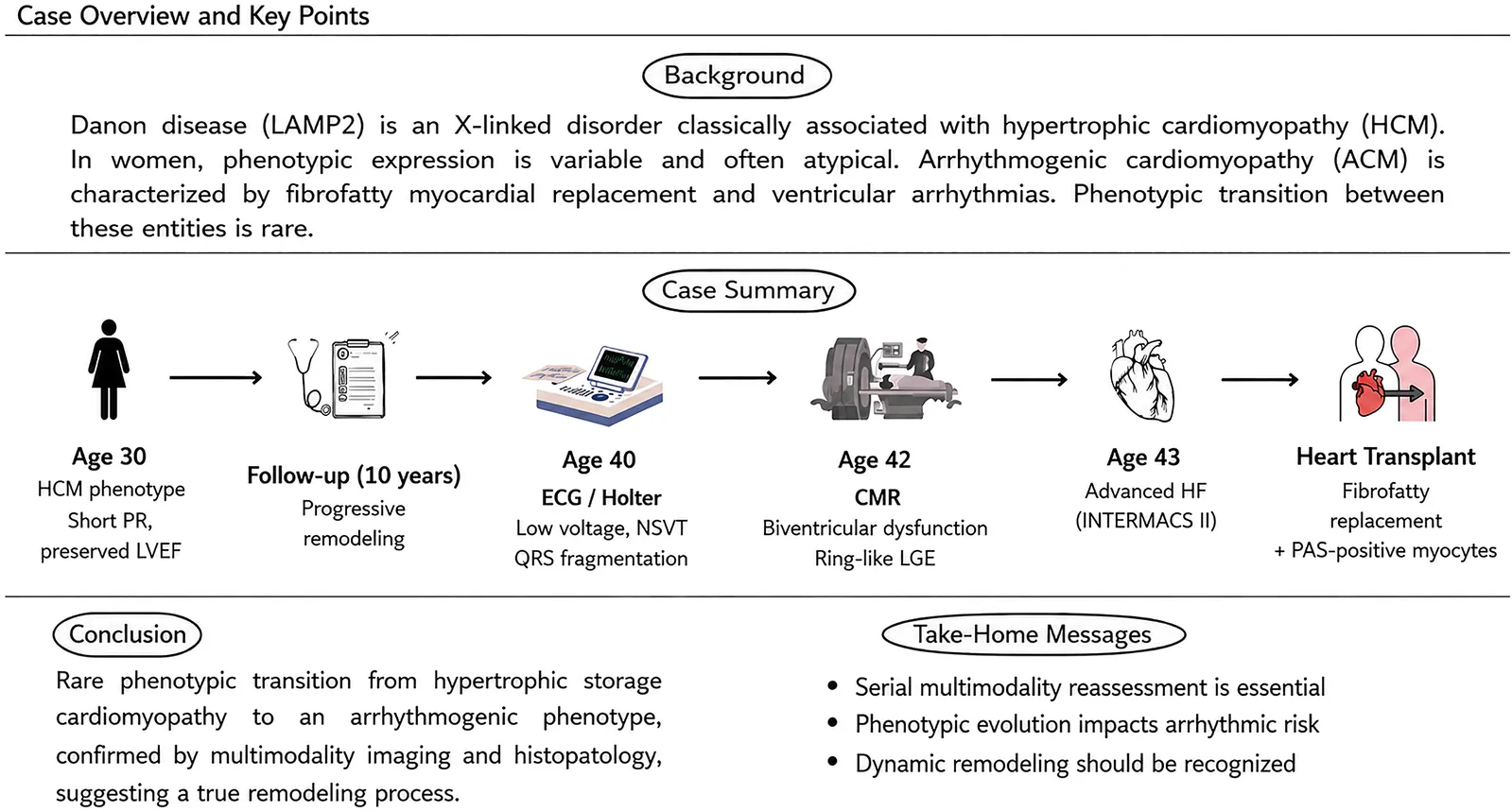
The Clinical Course in a Female Patient With Danon Disease ACM = arrhythmogenic cardiomyopathy; CMR = cardiovascular magnetic resonance; ECG = electrocardiogram; HCM = hypertrophic cardiomyopathy; HF = heart failure; INTERMACS = Interagency Registry for Mechanically Assisted Circulatory Support; LAMP2 = lysosome-associated membrane protein 2; LGE = late gadolinium enhancement; LVEF = left ventricular ejection fraction; NSVT = nonsustained ventricular tachycardia; PAS = periodic acid–Schiff.

## Discussion

Danon disease is a rare X-linked autophagic vacuolar cardioskeletal myopathy disorder caused by pathogenic variants in *LAMP2* and is classically associated with hypertrophic cardiomyopathy, skeletal myopathy, and conduction abnormalities.^1^ In women, the clinical presentation often is highly variable, presumably because of different degrees of X chromosome inactivation.^1^ The combination of low QRS complex voltages, fragmented depolarization, ventricular arrhythmias, right ventricular involvement, and fibrofatty replacement is suggestive of an arrhythmogenic remodeling process, confirmed by fibrofatty myocardial infiltration extending from epicardium toward the mid-myocardium in the explanted heart, consistent with ACM.^2^

The cardiac pathology of Danon disease is characterized by severe myocardial hypertrophy and accumulation of autophagic vacuoles containing glycogen, leading to progressive fibrosis,^3^ but not by fibrofatty replacement, which is a hallmark of ACM.^4^ This case illustrates an uncommon and clinically important phenotypic transition from an initial hypertrophic phenotype to ACM. The evolution was documented through serial multimodality assessment, including ECG, Holter monitoring, CMR, and histopathology. The combination of right ventricular involvement, high arrhythmic burden, ECG abnormalities (including low QRS complex voltage and fragmentation), and extensive subepicardial LGE fulfills contemporary diagnostic criteria for ACM according to the Padua criteria.^2^ Whether a pathogenic *LAMP2* variant can lead to a phenotype consistent with end-stage ACM remains an unresolved question. Fibrofatty replacement is a well-established mechanism mediated by plakoglobin within the beta-catenin signaling pathway, promoting a shift from myogenesis to adipofibrogenesis.^5^

Although very rare, arrhythmogenic phenotypes in women with LAMP2 disease have been reported in the literature. A 28-year-old woman first presented with a sustained ventricular arrhythmia as the first clinical manifestation.^6^ ECG showed sinus bradycardia and first-degree atrioventricular block, but unlike our case, the echocardiography revealed an enlarged right ventricular outflow tract and right atrial thrombosis.^6^ In a case report of a Sardinian family with a history of 4 sudden cardiac deaths, a woman initially presented with a transient ischemic attack at the age of 35, atrial fibrillation, ventricular pre-excitation on ECG, and mild concentric hypertrophy (13 mm).^7^ She developed ventricular dilatation and dysfunction (LVEF 25%) and received a biventricular defibrillator that interrupted a ventricular fibrillation episode.^7^ At age 48, she developed advanced heart failure requiring HTX, and only post-HTX was the genetic test performed and revealed a pathogenic variant (c.103_104delGA; p.Asp35Phefs∗20) in the *LAMP2* gene.^7^ In another reported family, the mother of a proband with genetically confirmed Danon disease presented with sustained ventricular arrhythmia at 32 years of age, requiring catheter ablation and an implantable cardioverter-defibrillator. Despite initial rhythm control, she exhibited rapid disease progression, developing advanced heart failure by age 35, which led to HTX, and she died because of perioperative complications.^8^ These presentations are noteworthy and resemble the emerging spectrum of *LAMP2* cardiomyopathy manifesting with prominent arrhythmogenic features in women rather than a purely hypertrophic phenotype, thereby broadening the recognized phenotypic spectrum of this disorder.

In our case, the woman initially presented with a hypertrophic phenotype and later developed an arrhythmogenic phenotype. Pathology demonstrated features of Danon disease but also the fibrofatty replacement. It may be hypothesized that *LAMP2* may exert an indirect effect on the beta-catenin pathway. However, there is currently no evidence to support this hypothesis yet.

Alternatively, the patient may harbor an additional, as yet unidentified, genetic cause of ACM. However, no additional pathogenic or likely pathogenic variants were identified on the Next-Generation Sequencing panel, and there was no family history suggestive of ACM. Although pathogenic variants are identified in only approximately 50% of patients with ACM, precluding complete exclusion of a second genetic disorder, it is equally plausible that the arrhythmogenic phenotype reflects progressive cardiomyocyte cell death and replacement fibrosis resulting from dysregulated autophagy in Danon disease.

## Conclusions

This case demonstrates a rare phenotypic transition in *LAMP2* cardiomyopathy, progressing from an initial hypertrophic storage phenotype to an ACM phenotype. This phenotype switch underscores the importance of repeated phenotypic reassessment with serial multimodality evaluation in surveillance of patients with genetic cardiomyopathies, with potential implications for arrhythmic risk stratification and family screening.

**Take-Home Message**

• Longitudinal multimodality reassessment is essential in genetic cardiomyopathies, as phenotypes may evolve over time and influence arrhythmic risk stratification and family screening.

## Funding Support and Author Disclosures

The authors have reported that they have no relationships relevant to the contents of this paper to disclose.
